# Is Sociodemographic Status Associated with Empathic Communication and Decision Quality in Diabetes Care?

**DOI:** 10.1007/s11606-021-07230-5

**Published:** 2022-01-01

**Authors:** Brigida A. Bruno, Karen Guirguis, David Rofaiel, Catherine H. Yu

**Affiliations:** 1 Faculty of Medicine, University of Toronto, Toronto, ON Canada; 2 Department of Family and Community Medicine, University of Toronto, Toronto, ON Canada; 3 University of Western Ontario, London, ON Canada; 4 The National University of Ireland, Galway, Galway, Ireland; 5 Division of Endocrinology and Metabolism, St. Michael’s Hospital, Toronto, ON Canada; 6 Dalla Lana School of Public Health, University of Toronto, Toronto, Canada; 7 Li Ka Shing Knowledge Institute, St. Michael’s Hospital, Toronto, ON Canada; 8 Department of Medicine, Faculty of Medicine, University of Toronto, Toronto, Canada

## Abstract

**Objective:**

To assess the relationship between empathic communication, shared decision-making, and patient sociodemographic factors of income, education, and ethnicity in patients with diabetes.

**Research Design and Methods:**

This was a cross-sectional study from five primary care practices in the Greater Toronto Area, Ontario, Canada, participating in a randomized controlled trial of a diabetes goal setting and shared decision-making plan. Participants included 30 patients with diabetes and 23 clinicians (physicians, nurses, dietitians, and pharmacists), with a sample size of 48 clinical encounters. Clinical encounter audiotapes were coded using the Empathic Communication Coding System (ECCS) and Decision Support Analysis Tool (DSAT-10).

**Results:**

The most frequent empathic responses among encounters were “acknowledgement with pursuit” (28.9%) and “confirmation” (30.0%). The most frequently assessed DSAT components were “stage” (86%) and knowledge of options (82.0%). ECCS varied by education (*p*=0.030) and ethnicity (*p*=0.03), but not income. Patients with only a college degree received more empathic communication than patients with bachelor’s degrees or more, and South Asian patients received less empathic communication than Asian patients. DSAT varied with ethnicity (*p*=0.07) but not education or income. White patients experienced more shared decision-making than those in the “other” category.

**Conclusions:**

We identified a new relationship between ECCS, education and ethnicity, as well as DSAT and ethnicity. Limitations include sample size, heterogeneity of encounters, and predominant white ethnicity. These associations may be evidence of systemic biases in healthcare, with hidden roots in medical education.

**Supplementary Information:**

The online version contains supplementary material available at 10.1007/s11606-021-07230-5.

## INTRODUCTION

Despite advances in medicine, social and economic factors contribute to 50% of a population’s health status^1^ (Canadian Institute for Advanced Research, Health Canada, 2002), and some estimate that less than 10–15% of mortality is preventable by medical care, with the remainder being attributed to social factors^2^. For example, citizens living in the 1% or 5% highest income counties in the USA had better health outcomes compared to average US citizens^3^. In contrast, those with lower socio-economic status had a greater prevalence of psychological and chronic health conditions^4^. These health disparities arise from historical inequities that result in decreased access to clean environments, housing, quality nutrition, and health care. This in turn predisposes to chronic stress and chronic disease development^5^.

Patient-centered care is a vital component of health care that improves the physical and psychosocial well-being of patients^6^. Shared decision-making, a component of patient-centered care^6^, involves assessing the patient’s decision-making needs, providing individualized support and evaluating patient goals to arrive at a quality decision informed by evidence and patients’ values and preferences^7^. Shared decision-making is associated with improved patient satisfaction and engagement in care^8^. Shared decision-making is facilitated by empathic communication^9^; it encompasses the cognitive capacity to understand a patient’s needs, an affective sensitivity to the patient’s feelings, and a behavioral ability to convey this to the patient^10^. A 2002 meta-analysis of medical interactions in primary care demonstrated that increased physician empathy was associated with increased patient satisfaction, adherence, comprehension, and perception of a good interpersonal relationship^11^.

Diabetes is a complex chronic disease that disproportionately affects racialized groups and those of lower socioeconomic status worldwide, with these groups experiencing increased prevalence, lower life expectancy, and increased complications of diabetes^5^. For example, in the USA, the risk of type 2 diabetes is 66% higher in Hispanic people and 77% higher in Black people^12^ than white people. In Canada, Indigenous people are three to five times more likely to have type 2 diabetes than non-Indigenous people^13^. Both low income and education are also associated with increased prevalence of diabetes: individuals with lower income and education are two to four times more likely to develop diabetes than more advantaged individuals^14^. In those with diabetes, low socioeconomic status was associated with a two-fold greater risk of all-cause, cardiovascular- and diabetes-related death compared to high-income counterparts^15^. A recent population-based study in the UK by Riley et al. (2021) showed that social deprivation is an independent risk factor for developing diabetic foot disease and related complications^16^.

Health disparities also exist in the receipt of patient-centered care, which may then worsen care gaps. National survey data demonstrate that racialized low-income patients in the USA perceive that they receive less patient-centered care, including less shared decision-making, trust and empathic communication, and as a result are less satisfied with their care^17^. Similarly, people living in areas of high deprivation (i.e., low income and education) in Scotland perceive their physicians as less empathic and had less desire for shared decision-making^18^. A survey study found that physicians viewed Black patients and patients of low and middle socioeconomic status as less intelligent, less rational, and less likely to adhere to medical advice or follow-up, than White and high socioeconomic status patients^19^. Additional studies using audiotapes and videotapes of patient-clinician encounters, as well as patient self-report, demonstrate that clinicians exhibit less empathic and participatory communication (characterized by information sharing and patient involvement in discussion) towards racialized and low income patients^20^. These implicit biases impact physicians’ communication and clinical decision-making^19,20^, and may impact patient outcomes.

Specific to diabetes care, clinician empathy has been associated with increased patient satisfaction, quality of life, reduced HbA1c, LDL cholesterol, and fewer diabetes complications^21^. However, little is known about the relationship between social determinants of health, clinician empathy, and shared decision-making in a diabetes-specific population.

Thus, we sought to quantify the relationship between empathic communication, shared decision-making, and sociodemographic factors of income, education, and ethnicity, using validated scales. Our primary objective was to evaluate the relationship between empathic communication and patient education, income, and ethnicity in individuals with diabetes attending primary care clinics in the Greater Toronto Area. The secondary objective was to evaluate the relationship between shared decision-making and patient education, income, and ethnicity in this same population. We hypothesized that patients with lower education, income and from ethnic minorities would experience less empathic communication and shared decision-making compared to those with high education, income, and patients who identified as white.

Specifically, empathic communication will be quantified by the Empathic Communication Coding System (ECCS)^10^ — an observer-rated measure of empathy, which has not been studied in this context before. Shared decision-making will be quantified by with the Decision Support Analysis Tool-10 (DSAT-10)^22^ — an observer-rated measure of a clinician’s ability to engage a patient in shared decision-making.

## METHODS

### Overview and Study Design

This is a cross-sectional study and secondary analysis of clinical encounter transcripts from a large randomized controlled trial that evaluated the impact of interprofessional shared decision-making tools for patients with diabetes and other comorbidities, on decisional conflict^23^. Sample size was based on 48 available audiotapes from the original study. We reported according to Strengthening the Reporting of Observational Studies (STROBE) guidelines for a cross-sectional study (Supplemental Table 1), with details on the original study and recruitment published elsewhere^24^.

### Settings and Participants

The previous study was a 10-site cluster randomized-controlled trial^25^. The trial recruited 53 clinicians from primary care practice groups across the GTA, one of the most multicultural cities in the world, where 51.5% of the city belonged to a visible minority^26^. Within each consenting clinician’s practice, patients 18 years of age or older, with diabetes and 2 other comorbidities were randomly selected and invited to participate in a study using a web-based goal-setting and shared decision-making aid via telephone, with a total of 213 patients included. Exclusion criteria included those who did not speak English, had documented cognitive deficits, were unable to provide consent, had limited life expectancy (<1 year), or were not available for a follow-up. In order to assess intervention fidelity during the trial (that is, how was MyDiabetesPlan used during the encounter), 48 clinical encounters were audio-recorded then transcribed. This constituted the data source for the current study.

### Study Outcomes

The primary outcome was empathic communication, measured using ECCS. The secondary outcome was shared decision-making, measured using DSAT-10.

### Data Sources

We used patient-reported sociodemographic information and transcripts of clinical encounters. Patients self-reported their ethnicity, education, and income through an online or mailed survey at the start of the prior trial. In the original study, clinical encounters were audiotaped for qualitative analysis to inform future iterations of the shared decision-making intervention. Of note, the prior study also consisted of patient questionnaires for patient-reported outcomes of decisional conflict, diabetes distress, assessment of care, and quality of life; however, these were not used in the present study.

### Data Collection Tools

#### Assessment of Empathic Communication

We conducted qualitative coding of the clinical encounter transcripts to derive a score for empathic communication, using ECCS. ECCS is an observer-rated measure of clinician empathy that measures empathy by examining clinician empathic responses to patient-created opportunities, with responses subsequently categorized on a scale from 0 (denial) to 6 (shared experience) (Supplemental Table 2). Because it is observer-rated, it eliminates biases associated with self-report.

#### Assessment of Decision Quality

We conducted qualitative coding of the clinical encounter transcripts to derive a score for decision quality using DSAT-10. The DSAT-10 evaluates the clinician’s ability to address the status of the decision; the patient’s knowledge of the options, benefits, and harms; the patient’s values and preferences associated with the decision; assessment of the involvement of others; and the patient’s preferred role in the decision-making and the next steps^22^. The DSAT-10 scale ranges from 0 to 10, with higher scores indicating more decisional support during the patient-clinician interaction (Supplemental Table 2). Because it is observer-rated, it eliminates biases associated with self-report.

### Data Analysis

Audio-recordings of the clinical encounters were transcribed verbatim and coded independently using ECCS and DSAT by two team members with expertise in qualitative coding. The first 20 transcripts were double coded until an inter-rater agreement of 75% was attained. Coders were blinded to participant characteristics.

We then calculated the weighted average empathy score from the ECCS and the total score from the DSAT-10. The unit of analysis was the transcript, so the average empathy score was calculated by dividing the total score by the number of empathic opportunities per encounter. The DSAT-10 score was reported as a total score out of 10. We a priori selected to evaluate the relationship between sociodemographic factors of education, income, and ethnicity with ECCS and DSAT-10.

### Statistical Methods

We used descriptive statistics to describe the characteristics of participants (patients and clinicians) and clinical encounters. For the primary outcome, we used one-way ANOVA to examine the effect of the categorical independent variables (income and education) entered as between-subjects factors, on our continuous dependent variable ECCS. If there was a significant effect of any factor, we conducted exploratory post hoc Tukey’s to determine which groups differed from each other^27^. For ethnicity (white vs. non-white; binary outcome), we used 2 independent sample *t*-test. For the secondary outcome, we used the Kruskal-Wallis test to examine the effect of the categorical independent variables (income and education) on our ordinal dependent variable DSAT^28^. If there was a significant effect of any factor, we conducted exploratory post hoc Dunn tests to determine which groups differed from each other^27^. For ethnicity, we used Mann-Whitney test. We conducted a Benjamini-Hochberg procedure to correct for multiple comparisons, a moderate false discovery rate of 0.15, given the exploratory nature of this study^29^. All analyses were done using SPSS Statistics for Windows, Version 20.0^30^.

## RESULTS

### Characteristics of Patients, Clinicians, and Clinical Encounters

We analyzed a total of 48 clinical encounters, involving 30 unique patients and 23 unique clinicians. Sociodemographic characteristics of patients and clinicians are indicated in Table 1. There were 26 male patients (54%) and 22 female patients (46%). The majority of patients were within the age ranges of 65–74 years old (50%) and retired (64%), with annual income >$60,000 (53%). The sample was primarily white (69%), with income and education being fairly uniform among patient demographics. All patients had type 2 diabetes, with the exception of 1 nonrespondent. Clinicians consisted mostly of family physicians, 61% of which were female, and majority (48%) had more than 16 years of practice (Table 1).

**Table 1:** Sociodemographic Characteristics of Patients (*n*=48) and Clinicians (*n*=23)

**Patient characteristic**	**Category**	Number* (% of total)
**Age**	45–54 years	2 (4.2)
	55–64 years	9 (18.8)
	65–74 years	24 (50.0)
	75–84 years	10 (20.8)
	85+ years	3 (6.3)
**Gender**	Male	26 (54.2)
	Female	22 (45.8)
**Employment**	Retired	30 (63.8)
	Full time/part time	11 (23.4)
	Unemployed	2 (4.3)
	Other	4 (8.5)
**Income**	<$30 K	10 (25.6)
	$30–59 K	8 (20.5)
	$60–100 K	10 (25.6)
	> $100 K	11 (28.2)
**Education**	High school or less	15 (31.9)
	College	16 (34.0)
	Bachelor’s or greater	16 (34.0)
**Ethnicity**	Chinese	5 (10.4)
	South Asian	6 (12.5)
	White/Caucasian	33 (68.8)
	Filipino	1 (2.1)
	Black Caribbean/African/African Canadian	2 (4.2)
	Aboriginal	1 (2.1)
**Clinician characteristic**	**Category**	Number* (% of total)
**Profession**	Family physician	15 (65.2)
	Registered nurse	5 (21.7)
	Registered dietician	2 (8.7)
	Pharmacist	1 (4.4)
**Gender**	Female	14 (60.9)
	Male	9 (39.1)
**Number of years in clinical practice**	<5	6 (26.1)
	6–10	3 (13.0)
	11–15	3 (13.0)
	≥ 16	11 (47.8)

*Total n for each characteristic may vary based on unspecified data for some individuals

Clinical encounters ranged in length from 3 min 55 s to 1 h 30 min, with a mean length of 31 min 26 s (SD 15 min 33 s). Mean ECCS score across all clinical encounters was 3.5 (standard deviation (SD) = 0.8). The most frequent empathic responses were “acknowledgement with pursuit” (29 %) and “confirmation” (30.0%) (Table 2). Mean DSAT score across all clinical encounters was 3.9 (SD = 1.8). The most frequently assessed DSAT components were the stage of decision-making (present in 86% of encounters) and intervening to provide the knowledge of options (present in 82% of encounters). The least frequently assessed DSAT components were assessing and intervening regarding the preferred role of the patient (present in 16% and 14% of encounters respectively) (Table 3).

**Table 2:** Relative Proportion of Each Empathic Response Across All Encounters (*n* =48)

**Empathic response**	***n*** **(%)**
Denial 0	26 (5.2%)
Perfunctory 1	56 (11.2%)
Implicit 2	42 (8.4%)
Acknowledgement 3	71 (14.2%)
Acknowledgement and pursuit 4	144 (28.9%)
Confirmation 5	148 (30.0%)
Shared feeling or experience 6	12 (2.4%)

**Table 3:** Frequency of DSAT Components Assessment Across All Encounters (*n*=48)

**DSAT components**	***n*** **(%)**	
**Uncertainty**	21 (42.0%)	
**Timing**	21 (42.0%)	
**Stage**	43 (86.0%)	
**Knowledge**	**Assess**	**Intervene**
Options	14 (28.0%)	41 (82.0%)
Benefits	9 (18.0%)	38 (74.0%)
Harms	11 (22.0%)	32 (64.0%)
**Values/preferences**		
Importance of benefits	30 (60.0%)	
Importance of harms	15 (30.0%)	
**Other’s involvement**		
Preferred role	8 (16.0%)	7 (14.0%)
Support	37 (74.0%)	27 (54.0%)
**Next steps**	38 (76.0%)	

*DSAT* Decision Support Analysis Tool

### Relationship Between Patient Sociodemographic Factors and Empathic Communication (Primary Outcome)

We found that ECCS was varied by education (*p*=0.030) and ethnicity (*p*=0.030), but not income (Table 4). Post hoc analyses of the former revealed that patients with only a college degree received more empathic communication than patients with bachelor’s degrees or more, and South Asian patients received less empathic communication than Asian patients (Table 4).

**Table 4:** Relationship Between Sociodemographic Factors, ECCS and DSAT-10

ECCS (one-way ANOVA)					
Factor	Effect size: F-statistic	Degrees of freedom	*p*-value	Post hoc Tukey HSD test (95% CI)	*p*-value
Income	0.429	3.35	0.733	Not applicable	
Education	3.787	2.44	0.030	College > Bachelor+: 0.69 (0.01–1.36)	0.044
Ethnicity	3.001	4.43	0.029	Chinese > South Asian 1.60 (0.23 to2.98)	0.015
DSAT (Kruskal-Wallis)					
Factor	Effect size: eta^2^	Degrees of freedom	*p*-value	Post-hoc Dunn test (standard error)	*p*-value
Income	0.063	3	0.493	Not applicable	
Education	0.011	2	0.775	Not applicable	
Ethnicity	0.18	4	0.072	White > Other: 16.56 (6.60)	0.012

### Relationship Between Patient Sociodemographic Factors and Shared Decision-Making (Secondary Outcome)

We found that DSAT varied with ethnicity (*p*=0.07) but not education or income (Table 4). Post hoc analyses of the former revealed that white patients experienced more shared decision-making than those in the “other” category (Table 4).

To correct for multiple comparisons, we conducted a Benjamini-Hochberg procedure using a conservative false discovery rate of 0.15. The comparison between DSAT and patient ethnicity had the highest *P* (=0.07) that was less than its critical Benjamini-Hochberg value (0.08), thus confirming that all preceding comparisons (ECCS and ethnicity, ECCS and education) were significant. The rank table is included in Supplemental File 2.

## DISCUSSION

Our study demonstrated that ECCS was varied by education and ethnicity, such that patients with only a college degree received more empathic communication than patients with bachelor degrees or higher, and South Asian patients received less empathic communication than Asian patients. In addition, we found that DSAT varied by ethnicity, such that white patients received more shared decision-making than non-white patients/“other.”

### Interpretation of Findings/Relevance to Literature

These findings are consistent with the existing literature that racialized individuals and those with lower SES perceive less empathy and shared decision-making in healthcare interactions^31^. However, we found that patients with only college education received more empathic communication compared to those with a bachelor degree or higher, which reveals a new finding that is contrary to the overarching trend in the literature, despite controlling for income and ethnicity^32^. Although several studies have demonstrated that individuals with low income, education and from ethnic minorities experience less empathic communication and shared decision-making, we provide objectively assessed, quantitative evidence of these relationships, in a clinical population — individuals with type 2 diabetes — that is characterized by ethnic and socioeconomic diversity; these relationships existed despite the study being conducted in a multicultural geographic setting during an era with growing awareness of considerations for equity, diversity, and inclusion.

These associations are concrete evidence of systemic bias in healthcare, with roots in medical education. Empathy decreases throughout medical training^33^. Further, studies of medical trainees’ attitudes towards racialized populations have demonstrated a “pro-white” bias in empathic communication and treatment, in that medical trainees held false beliefs regarding black patients’ perception of pain which led to under-treatment^20^. This was confirmed in a systematic review by Hall and colleagues, which showed that healthcare providers have implicit biases in terms of negative attitudes towards people of color, which impacted patient-provider interactions, treatment decisions, and patient health outcomes^34^. Similarly, patients of lower socioeconomic status perceive less access to care, altered physician-patient interaction (feeling like they are listened to), and differences in management plan (such as reduced diagnostic testing)^35^. Taken together, strategies must be implemented at the medical education level to foster empathy and address these biases that are often part of the hidden curriculum. Examples of strategies to enhance empathic communication include assessing provider patient-centered communication at the point-of-care, education among peers, and mentorship by clinicians who score highly on patient-rated scales of patient-centered communication^36^. Strategies to address systemic bias in medical education include standardized anti-racism and anti-bias training^37^ as well as implementation of a structural competency framework, including improving recruitment, promotion and retention processes of faculty, “stop the line” processes for racism, and the use of a community council to review health equity initiatives and provide feedback on performance^38^.

### Strengths and Limitations

First, our study was limited by small predominantly white sample; however, this was a hypothesis-generating exploratory study wherein we a priori selected specific outcomes and statistically controlled for multiple comparisons. Second, because our findings were based on audio-recordings of clinical encounters alone, we were unable to assess non-verbal empathic communication, a key component of empathic communication^18^. Third, heterogeneity of appointment type (i.e., follow-up vs. initial appointments) may have resulted in differing levels of empathic communication and shared decision-making, given that shared decision-making is longitudinal in nature occurring over several appointments^8^. We tried to account for this heterogeneity by adjusting for the number of empathic opportunities per encounter; however, we could not adjust the overall DSAT score based on encounter length because of scale properties. Fourth, we did not examine the impact of clinician factors on empathy or shared-decision-making, including burnout, workload, gender, and training^32^, which may have influenced our results.

Study strengths include our use of objective third-party observer-rated scales not previously used in this context that reduce bias associated with patient or clinician self-report. Second, we captured representative clinical encounters with different clinicians, including physicians, nurses, and dietitians, consistent with interprofessional diabetes care.

## Next Steps and Implications

Our research has implications for medical education, clinical practice, and research. That empathy declines with medical training and that racial biases are prevalent in medical trainees call into question the adequacy of medical education in preparing physicians to care for patients with a lens of social justice. Several professional organizations have advocated for education interventions to prepare medical trainees to care for the needs of a culturally diverse population including cultural competency training^39^, and incorporating critical reflection and dialogue into curriculum to address biases and assumptions that shape healthcare interactions^40^. In terms of clinical implications, our research underscores the importance of clinical interventions such as shared decision-making tools to empower patients — in particular racialized individuals and those of lower income and educational attainment — to become involved in their healthcare. Additional supports such as interprofessional teams and peer coaches^36^ should be leveraged to enable patients in vulnerable groups to play an active role in their care. In terms of implications for research, future studies should confirm our findings, and specifically assess the trend of education level and empathy in a larger and more diverse patient population during chance encounters. Triangulation of objective rating of empathic communication (as in our study) with patient self-report of empathy as well as the patient lived experience using qualitative methodology would enhance our understanding. Future studies could also test the impact of patient-directed interventions (such as peer coaches) as well as clinician-directed interventions (such as professional development regarding implicit bias aimed at improving empathic communication or shared decision-making) in specific vulnerable populations.

## Supplementary Information

Below is the link to the electronic supplementary material.

Supplementary file1 (DOCX 21.1 KB)

Supplementary file2 (DOCX 15 KB)
