# Supplementary material for: Is Sociodemographic Status Associated with Empathic Communication and Decision Quality in Diabetes Care?
Source: J Gen Intern Med. 2022 Jan 1;37(12):3013–9. doi: 10.1007/s11606-021-07230-5 (PMC9485322; doi:10.1007/s11606-021-07230-5)
Supplement: Supplementary file 1 — Supplementary file1 (DOCX 21.1 KB) [file 11606_2021_7230_MOESM1_ESM.docx]

**Supplemental Files**

**Table 1: Rank Table for Benjamini-Hochberg Procedure to Correct for Multiple Comparisons with a False Discovery Rate of 0.15**

| Variable | p | rank | (i/m)Q |
| --- | --- | --- | --- |
| ECCS ethnicity | 0.024 | 1 | 0.025 |
| ECCS education | 0.025 | 2 | 0.05 |
| DSAT patient ethnicity | 0.072 | 3 | 0.075 |
| DSAT patient income | 0.493 | 4 | 0.1 |
| ECCS education | 0.742 | 5 | 0.125 |
| DSAT education | 0.78 | 6 | 0.15 |

**Supplemental Table 2**

STROBE Statement – Checklist of Items that Should be included in Reports of Cross-Sectional Studies

|  | **Item No.** | **Page No.** | **Recommendation** |
| --- | --- | --- | --- |
| **Title and Abstract** | 1 | 1 | *(a)* Indicate the study’s design with a commonly used term in the title or the abstract  *(b)* Provide in the abstract an informative and balanced summary of what was done and what was found |
| **Introduction** | | | |
| Background/Rationale | 2 | 2-4 | Explain the scientific background and rationale for the investigation being reported |
| Objectives | 3 | 5 | State specific objectives, including any prespecified hypotheses |
| **Methods** | | | |
| Study Design | 4 | 6 | Present key elements of study design early in the paper |
| Setting | 5 | 6 | Describe the setting, locations, and relevant dates, including periods of recruitment, exposure, follow-up, and data collection |
| Participants | 6 | 6 | *(a)* Give the eligibility criteria, and the sources and methods of selection of participants |
| Variables | 7 | 6-7 | Clearly define all outcomes, exposures, predictors, potential confounders, and effect modifiers. Give diagnostic criteria, if applicable |
| Data Sources/Measurement | 8* | 7 | For each variable of interest, give sources of data and details of methods of assessment (measurement). Describe comparability of assessment methods if there is more than one group |
| Bias | 9 | 7-8 | Describe any efforts to address potential sources of bias |
| Study Size | 10 | 6 | Explain how the study size was arrived at |
| Quantitative Variables | 11 | 8 | Explain how quantitative variables were handled in the analyses. If applicable, describe which groupings were chosen and why |
| Statistical Methods | 12 | 8-9 | (a) Describe all statistical methods, including those used to control for confounding  *(b)* Describe any methods used to examine subgroups and interactions  *(c)* Explain how missing data were addressed  *(d)* If applicable, describe analytical methods taking account of sampling strategy  *(e)* Describe any sensitivity analyses |
| **Results** | | | |
| Participants | 13* | 10 | (*a)* Report numbers of individuals at each stage of study—eg numbers potentially eligible, examined for eligibility, confirmed eligible, included in the study, completing follow-up, and analysed  *(b)* Give reasons for non-participation at each stage  *(c)* Consider use of a flow diagram |
| Descriptive Data | 14* | 10-11 | *(a)* Give characteristics of study participants (eg demographic, clinical, social) and information on exposures and potential confounders  *(b)* Indicate number of participants with missing data for each variable of interest |
| Outcome Data | 15* | 12-13 | Report numbers of outcome events or summary measures |
| Main Results | 16 | 13-14 | *(a)* Give unadjusted estimates and, if applicable, confounder-adjusted estimates and their precision (eg, 95% confidence interval). Make clear which confounders were adjusted for and why they were included  *(b)* Report category boundaries when continuous variables were categorized  *(c)* If relevant, consider translating estimates of relative risk into absolute risk for a meaningful time period |
| Other Analyses | 17 | 14-15 | Report other analyses done—eg analyses of subgroups and interactions, and sensitivity analyses |
| **Discussion** | | | |
| Key Results | 18 | 15-16 | Summarise key results with reference to study objectives |
| Limitations | 19 | 17-18 | Discuss limitations of the study, taking into account sources of potential bias or imprecision. Discuss both direction and magnitude of any potential bias |
| Interpretation | 20 | 16-17 | Give a cautious overall interpretation of results considering objectives, limitations, multiplicity of analyses, results from similar studies, and other relevant evidence |
| Generalisability | 21 | 18-19 | Discuss the generalisability (external validity) of the study results |
| **Other Information** | | | |
| Funding | 22 | N/A | Give the source of funding and the role of the funders for the present study and, if applicable, for the original study on which the present article is based. |

*Give information separately for exposed and unexposed groups
