# Supplementary material for: Is Sociodemographic Status Associated with Empathic Communication and Decision Quality in Diabetes Care?
Source: J Gen Intern Med. 2022 Jan 1;37(12):3013–9. doi: 10.1007/s11606-021-07230-5 (PMC9485322; doi:10.1007/s11606-021-07230-5)
Supplement: Supplementary file 2 — Supplementary file2 (DOCX 15 KB) [file 11606_2021_7230_MOESM2_ESM.docx]

**Table 1: Description and psychometric properties of ECCS and DSAT-10 (Supplemental)**

| **Scale** | **Number of Items** | **Description** | **Example** | **Psychometric Properties** |
| --- | --- | --- | --- | --- |
| **ECCS** | 7 Response Levels | A 7-level scale categorizing health care professional responses to patient-initiated empathic opportunities during their clinical encounter. Levels range from denial (0), perfunctory recognition (1), implicit recognition (2), acknowledgement (3), acknowledgement and pursuit (4), confirmation (5) and shared experience (6). | **Denial** (0)  Patient: “I’m really concerned about getting off of this medication.” Clinician: “Change into the gown now.”  **Perfunctory recognition** (1): Patient: “It has been a difficult week for me.”  Clinician: “Mhm.”  **Implicit recognition** (2):  Patient: “This headache makes it difficult for me to work”  Clinician: “How is the insurance business lately?”  **Acknowledgement** (3): Clinician: “Yes you mentioned that you’re feeling sad”  **Acknowledgement and pursuit** (4):  “You mentioned that you’re feeling sad, can you tell me more about what has been going on?”  **Confirmation** (5): “You sound like you are very busy, I see why it would be tough for you tine find time to exercise.”  **Shared experience** (6):  “I understand how scary this must be for you, my husband had a biopsy and we were really scared.” | Validated in patient initial visits to a general internal medicine clinic where patients created at least 1 explicit empathic opportunity (statistical power of at least .85 at p = .05). |
| **DSAT-10** | 5 categories | A 5-category coding system including decision-making status, knowledge, values/preferences, others’ involvement in the decision, and next steps, to evaluate the health-care professionals use of support and communication skills during a clinical encounter. | **Decision-making status** includes the clinician identifying uncertainty about the decision, timing for when the decision is to be made, and stage of decision-making.  **Knowledge** includes assessment of knowledge of options, benefits, and harms.  **Values and preferences** include discussion of the importance of benefits and harms.  **Others’ involvement** in the decision includes discussion of preferred role in decision making, others’ involvement and pressure or support from others.  **Next steps** involve summarizing next steps to address patients’ decision making needs. | Validated in standardized patients making health decisions (The kappa coefficient over all items was 0.55 (95% CI: 0.49, 0.61) with higher agreement for encounters involving trained nurses (0.62; 95% CI: 0.43, 0.80). |

ECCS: Empathic Communication Coding System, DSAT: Decision Support Analysis Tool.
